# Radicality and revision surgery in vestibular schwannoma treatment: balancing optimal outcomes with unavoidable necessity

**DOI:** 10.1007/s00405-026-10365-y

**Published:** 2026-06-10

**Authors:** Kaňa Martin, Vlasák Aleš, Bubeníková Adéla, Lazák Jan, Koucký Vladimír, Chovanec Martin, Betka Jan, Fík Zdeněk

**Affiliations:** 1 Department of Otorhinolaryngology, Head and Neck Surgery, First Faculty of Medicine, Charles University, University Hospital Motol and Homolka, Prague, Czech Republic; 2 Department of Neurosurgery, Second Faculty of Medicine, Charles University, University Hospital Motol and Homolka, Prague, Czech Republic; 3 Department of Otorhinolaryngology, Third Faculty of Medicine, Charles University, University Hospital Královské Vinohrady, Prague, Czech Republic

**Keywords:** Vestibular schwannoma, Revision surgery, Facial nerve reconstruction, Stereoradiotherapy

## Abstract

**Purpose:**

Revision microsurgery for vestibular schwannoma (VS) after failure of prior microsurgery and/or stereotactic irradiation represents a technically demanding scenario with uncertain functional outcomes. We aimed to evaluate operative strategy, extent of resection, tumor control, and functional results—primarily facial nerve outcomes—in patients undergoing revision VS surgery in a high-volume tertiary center.

**Methods:**

A retrospective single-center cohort study included consecutive patients who underwent revision VS microsurgery between 1998 and 2025. Demographic, radiological, surgical, and audiological data were analyzed. Primary endpoint was long-term facial nerve function assessed by House–Brackmann (HB) grading. Secondary endpoints included extent of resection, tumor control on follow-up MRI, hearing outcome, and perioperative morbidity.

**Results:**

Nineteen patients (2.6% of 741 treated VS cases) underwent revision surgery. Most tumors were advanced (Koos grade 4, 74%). Gross total resection was achieved in 79%. Immediate postoperative HB VI occurred in 58%, improving over time to 11% at last follow-up. Favorable long-term facial function (HB I–III) was observed in 68%. Hearing preservation was not achieved. Perioperative complications occurred in 42%, most commonly cerebrospinal fluid pseudocyst. Radiological tumor control reached 95% during a mean follow-up of 136 months.

**Conclusion:**

Revision VS microsurgery provides high rates of tumor control with acceptable facial nerve outcomes but limited hearing preservation. It should be reserved for carefully selected patients and performed in experienced centers with cranial nerve reconstruction expertise.

## Introduction

Vestibular schwannoma (VS) is a benign, typically slow-growing tumor of the vestibular division of the vestibulo-cochlear nerve. Over recent decades, the therapeutic paradigm has shifted from primarily maximizing extent of resection toward a risk-adapted strategy that prioritizes functional preservation (most notably facial nerve function, hearing and health-related quality of life) alongside durable tumor control [1]. This shift has been enabled by improved characterization of the natural history of VS and by advances in microsurgical technique, intraoperative neurophysiological monitoring, and stereotactic irradiation (e.g. wait and rescan strategy protocols for selected patients) [2, 3]. When active treatment is indicated due to documented growth, symptom progression, or mass effect, the choice between microsurgical resection and stereotactic irradiation (stereotactic radiosurgery [SRS] and/or stereotactic radiotherapy [SRT]) is guided by tumor size and configuration, growth kinetics, baseline cranial nerve status, patient age and comorbidities, and institutional expertise.

In microsurgery, outcome reporting increasingly emphasizes standardized functional metrics (e.g., House–Brackmann [HB] grading for facial nerve function, AAO-HNS for hearing class) and complication profiles rather than extent of resection alone. Concurrently, there is consistent evidence for a volume–outcome relationship in complex skull base surgery, supporting centralization of VS microsurgery to high-volume tertiary centers and the use of national registries to monitor quality and enable benchmarking (e.g., BANA in the United Kingdom) [4–6].

Despite generally favorable primary outcomes, treatment failure remains clinically relevant. After microsurgery, recurrence or regrowth risk is low but not negligible and increases with longer follow-up and less-than-gross total resection, while reported rates vary substantially due to heterogeneous definitions (recurrence vs. regrowth), imaging protocols, and follow-up duration (e.g. reported radiological recurrence rates after gross-total resection range from 4% at 5 years to 18% at 10 years in long-term historical cohorts, compared with regrowth rates of 53% and 83% after subtotal resection) [7]. After stereotactic irradiation, tumor control is high in most series, yet progression can occur and reported control rates span a broad range (approximately 69%–97%), reflecting differences in patient selection (irradiation of primarily non-growing tumours), dose/fractionation, and criteria for progression depending of the growth speed [8, 9]. Moreover, a combined strategy—planned subtotal/near-total resection followed by adjuvant irradiation of residual tumor—has been proposed to balance functional preservation and tumor control, but remains debated because long-term control and the downstream consequences of salvage interventions are not fully characterized [10]. Importantly, microsurgery in skilled hands can achieve results comparable to stereoradiotherapy itself [11].

Management is most challenging in patients with prior treatment failure, including recurrence/regrowth after microsurgery and radiological progression or symptomatic deterioration after stereotactic irradiation. Revision microsurgery in this setting is technically demanding due to fibrosis, obliterated arachnoid planes, altered anatomy, and increased adherence of tumor to cranial nerves, brainstem, and vascular structures. These factors can increase operative complexity and morbidity, particularly compromising the probability of favorable facial nerve outcome, and may necessitate nerve repair or reanimation strategies. However, contemporary evidence on revision surgery is limited by small cohorts, heterogeneous inclusion criteria, and variable endpoint definitions, leaving uncertainty regarding expected tumor control, facial nerve outcomes, and complication rates across the main clinical scenarios (post-microsurgery vs. post-irradiation failure).

The objective of the present study is to report our single-center experience with revision microsurgery for vestibular schwannoma after failed stereotactic irradiation and/or previous microsurgical treatment. Specifically, we aim to (i) describe operative strategy and technical considerations in revision cases, (ii) quantify extent of resection and subsequent tumor control, and (iii) evaluate functional outcomes—primarily facial nerve function and secondarily hearing—together with perioperative morbidity in a consecutive cohort treated in a high-volume tertiary setting. Through these endpoints, we seek to better delineate the role, risks, and realistic expectations of revision VS surgery within contemporary outcome-focused management.

## Materials and methods

### Study design and and patient selection

We performed a single-center retrospective cohort study of patients who underwent revision microsurgery for VS at a tertiary referral center between 1998 and June 2025. Patients were identified using institutional surgical databases. Clinical, operative, radiological, and audiological data were extracted from electronic medical records, operative reports, imaging archives, and formal audiology documentation.

Patients were eligible if they met all of the following: (i) diagnosis of vestibular schwannoma confirmed histopathologically at revision surgery; (ii) at least one prior active treatment modality (microsurgery and/or stereotactic irradiation); (iii) availability of operative documentation and baseline imaging sufficient to classify tumor stage and EOR. On the other hand, patients were excluded if they had: (i) non-VS pathology, or (ii) insufficient documentation to determine prior treatment modality and indication for revision.

Patients were categorized by the modality preceding revision surgery: (i) prior microsurgery alone; (ii) prior microsurgery plus stereotactic irradiation; (iii) prior stereotactic irradiation alone (Gamma Knife or LINAC; SRS/SRT).

### Definitions and study endpoints

Revision surgery was defined as any subsequent microsurgical resection performed after a prior active treatment for VS (microsurgery and/or stereotactic irradiation) due to radiologically documented tumor recurrence or regrowth and/or clinically significant progression.

The primary endpoint: facial nerve functional outcome assessed by the HB grading system at last available follow-up (and additionally at predefined time points when available, e.g., discharge and 12 months). Secondary endpoints were: (i) extent of resection (EOR) at revision surgery; and (ii) tumor control after revision surgery, defined as absence of radiological progression on follow-up MRI. Additional outcomes were perioperative morbidity and cranial nerve–related complications after revision surgery, and hearing outcome (audiometric and AAO-HNS class).

Recurrence was defined as new tumor growth after an intended gross total resection (GTR) on baseline postoperative MRI. Regrowth was defined as increase in residual tumor volume/size after subtotal/near-total resection. For patients previously treated with stereotactic irradiation, post-irradiation progression was defined as sustained radiological growth on serial MRI beyond expected transient post-treatment changes (pseudoprogression), according to institutional criteria (≥ 2 mm increase on two consecutive MRIs ≥ 6 months apart).

### Data collection

Demographic and clinical variables were extracted from institutional records, including patient age at revision surgery, sex, and tumor laterality. Details of the index (primary) treatment were recorded, comprising treatment modality, surgical approach, documented extent of resection, pre-treatment tumor dimensions along three orthogonal axes, and the time interval from the index treatment to radiological recurrence or regrowth prompting revision. Clinical status at recurrence/regrowth was captured with emphasis on otological and neurological symptomatology, including hearing status classified according to the AAO–HNS system, tinnitus, vestibular symptoms (imbalance/vertigo), headache, and other cranial nerve deficits. Radiological tumor characteristics at recurrence/regrowth were classified using the Koos grading system and the Hannover (INT) classification, and the presence of cystic degeneration was documented. For the revision procedure, surgical approach, extent of resection, intraoperative facial nerve status, and facial nerve reconstruction technique (when applicable) were recorded. Postoperative outcomes included facial nerve function, hearing preservation, and perioperative complications.

### Statistical analysis

Descriptive statistics were used to summarize patient characteristics and surgical outcomes. Continuous variables are presented as means with ranges, categorical variables as counts and percentages.

## Results

### Cohort overview

Between January 1998 and June 2025, a total of 741 patients with sporadic VS were treated at our institution. Nineteen patients (2.6%) underwent revision microsurgery for radiologically documented recurrence/regrowth or treatment failure. Based on the treatment modality preceding revision, 12 patients (63%) were revised after primary microsurgery, 2 patients (11%) after combined microsurgery and stereotactic irradiation (Gamma Knife in both cases), and 5 patients (26%) after stereotactic irradiation alone (Gamma Knife in 4 cases and LINAC in 1). Histopathological examination confirmed VS in all cases.

The cohort consisted of 8 men (42.1%) and 11 women (57.9%), with a mean age at revision surgery of 48.5 years (range 28–68 years). Tumor laterality was nearly evenly distributed (10 left-sided, 9 right-sided). Mean follow-up after revision surgery was 136 months (range 31–300 months). Within our institutional primary microsurgical cohort (*n* = 722), 8 patients underwent revision surgery, corresponding to a revision rate of 1.1%. The remaining revision cases included patients who had undergone primary microsurgical treatment at external institutions and were subsequently referred to our department for revision surgery. Key cohort characteristics are summarized in Table 1.

**Table 1:** Cohort overview

Characteristic	Value
Study period	Jan 1998 – Jun 2025
Revision microsurgery cases	19 (2.6% of 741)
Sex	8 men (42.1%), 11 women (57.9%)
Age at revision, years	mean 48.5 (range 28–68)
Laterality	10 left (52.6%), 9 right (47.4%)
Follow-up after revision, months	mean 136 (range 31–300)
Institutional revision rate within primary microsurgery cohort	8/722 (1.1%)

### Primary treatment characteristics

Among patients with prior microsurgery (14/19, 73.7%), the index approach was predominantly retrosigmoid (13/14, 92.9%), with translabyrinthine in one case. The extent of resection at the index microsurgery was reported as GTR in 6/14 (42.9%) and STR in 8/14 (57.1%), most commonly due to adherence of the tumor to critical neurovascular structures or concern regarding neurological morbidity. The mean interval from index therapy to revision was 63.2 months (range 9–150). Clinical presentation at revision reflected substantial morbidity: vestibular complaints (11/19, 57.9%) and tinnitus (9/19, 47.4%) were frequent; preoperative facial nerve dysfunction was present in 6/19 (31.6%), and trigeminal symptoms in 4/19 (21.1%). At presentation, 16/19 patients (84.2%) were deaf on the treated side; among the three patients with preserved hearing, only one had serviceable hearing (AAO-HNS class A–B).

### Tumor characteristics and revision surgery

Tumors were predominantly advanced at revision: Koos grade 4 accounted for 14/19 cases (73.7%), including one Koos grade 4b lesion with hydrocephalus. Hannover (INT) staging was most commonly classified as T3 (10/19, 52.6%) and T2 (5/19, 26.3%). Cystic degeneration was observed in 8/19 (42.1%).

Revision resection was performed mainly via a retrosigmoid–transmeatal approach (16/19, 84.2%); a translabyrinthine approach was used in 1/19 (5.3%) and a combined retrosigmoid plus translabyrinthine strategy in 2/19 cases (10.5%), both performed as staged procedures. GTR was achieved in 15/19 patients (78.9%), NTR in 2/19 (10.5%), STR in 1/19 (5.3%), and partial resection in 1/19 (5.3%). Intraoperatively, facial nerve discontinuity was identified in 9/19 patients (47.4%); all underwent reconstruction (XII–VII anastomosis in 7 cases and interposition graft in 2). Tumor and surgical details are provided in Table 2.

**Table 2:** Distribution of tumor stage at revision according to Koos and Hannover (INT) classifications, presence of cystic degeneration, revision surgical approach, extent of resection (EOR), and intraoperative facial nerve findings including reconstruction technique. Values are reported as n (%)

Variable	*n* (%)
Koos grade	
1	1 (5.3)
2	2 (10.5)
3	2 (10.5)
4	14 (73.7)
Hannover (INT)	
T0 / T1 / T2 / T3 / T4	1 (5.3) / 2 (10.5) / 5 (26.3) / 10 (52.6) / 1 (5.3)
Cystic degeneration	8 (42.1)
Revision approach	
Retrosigmoid–transmeatal	16 (84.2)
Translabyrinthine	1 (5.3)
Combined retrosigmoid + translabyrinthine (staged)	2 (10.5)
Extent of resection at revision	
GTR / NTR / STR / partial	15 (78.9) / 2 (10.5) / 1 (5.3) / 1 (5.3)
Facial nerve discontinuity intraoperatively	9 (47.4)
Facial nerve reconstruction (*n* = 9)	XII–VII (Darrouzet) 7; interposition graft 2

Perioperative complications were observed in 8/19 patients (42.1%). The most frequent complication was cerebrospinal fluid pseudocyst formation occurring in 5 patients (26.3%; 2 revisions after stereotactic irradiation, 3 after primary microsurgery). Cerebellar dysfunction was documented in 2 patients, and transient lower cranial nerve (CN IX–XI) dysfunction occurred in 1 patient (5.3%). No case or perioperative mortality was recorded. Complications are summarized in Table 3.

**Table 3:** Perioperative complications following revision microsurgery for vestibular schwannoma, stratified according to the primary treatment modality preceding revision (primary microsurgery, combined microsurgery and stereotactic irradiation, or stereotactic irradiation alone). Values are reported as n (%)

Complication/Index therapy	Primary Stereotactic irradiation	Primary Microsurgery	Combined Treatment	Total
CSF pseudocyst	2 (50%)	3 (43%)	0	5 (38%)
Cerebellar dysfunction	0	1 (14%)	1 (50%)	2 (15%)
Transient CN IX–XI dysfunction	0	0	1 (50%)	1 (8%)

### Functional outcomes and tumor control

**Fig. 1:**
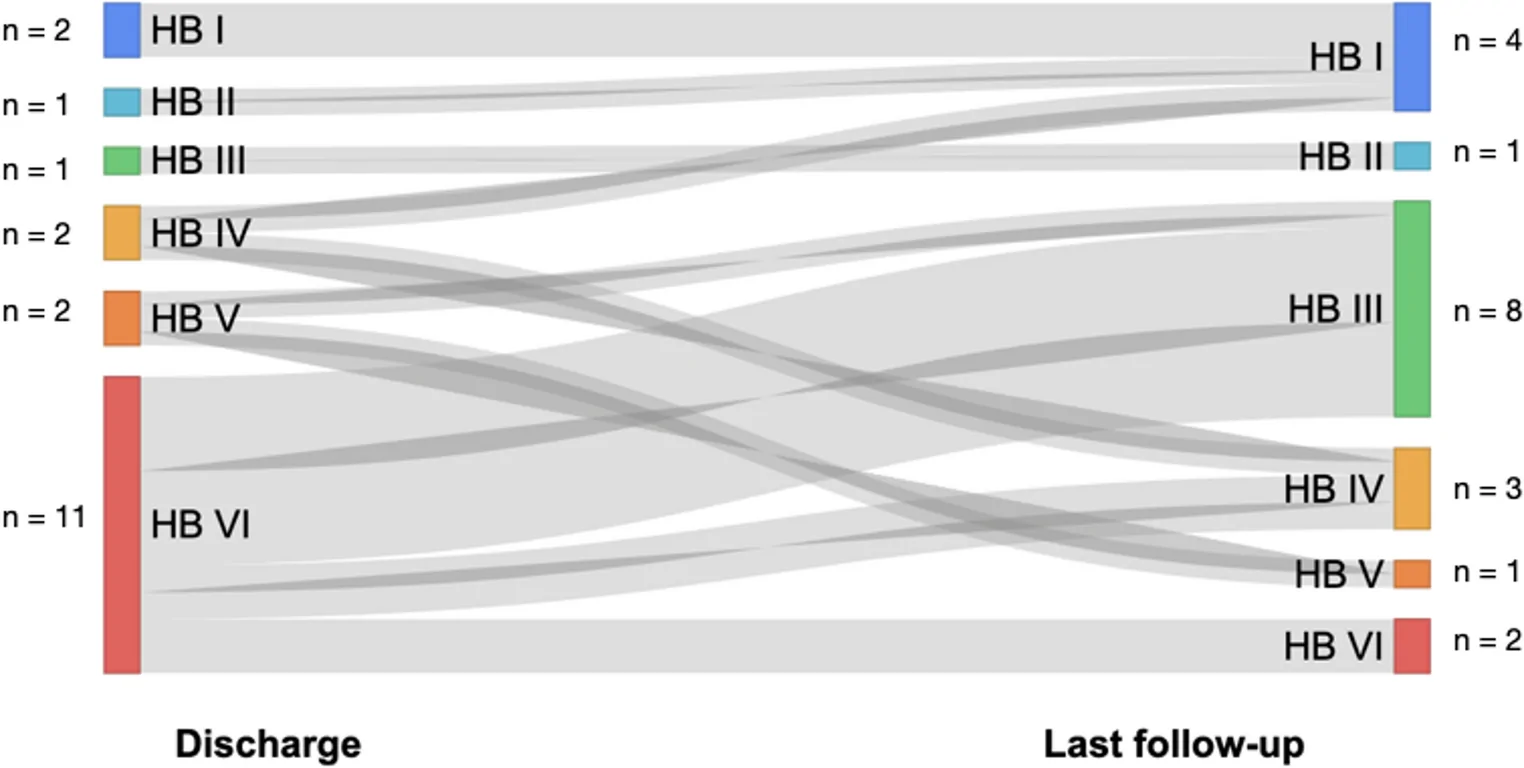
Sankey diagram depicting transitions in facial nerve function (HB) from hospital discharge to last follow-up after revision VS surgery. Ribbon width is proportional to the number of patients moving between HB grades. Node labels indicate HB grade at each time point, with corresponding patient counts shown (built in open-source R software)

Immediately after revision surgery, complete facial palsy (HB VI) was observed in 11/19 patients (57.9%). At last follow-up, persistent HB VI remained in 2/19 patients (10.5%, Fig. 1). Overall, a favorable long-term facial nerve outcome (HB I–III) was achieved in 13/19 patients (68.4%). The distribution of HB grades at discharge and last follow-up is shown in Table 4; Fig. 1. Hearing preservation was not achieved in any patient following revisionsurgery.

**Table 4:** House–Brackmann (HB) facial nerve function at discharge and at last follow-up, rates of favorable long-term facial outcome (HB I–III), complete palsy (HB VI) early and at last follow-up, hearing preservation after revision, and tumor control during follow-up. Values are reported as n (%) unless otherwise specified

Outcome	Value
HB at discharge (I / II / III / IV / V / VI)	2 / 1 / 1 / 2 / 2 / 11
HB at last follow-up (I / II / III / IV / V / VI)	4 / 1 / 8 / 3 / 1 / 2
Favorable long-term facial outcome (HB I–III)	13/19 (68.4%)
HB VI immediately post-op	11/19 (57.9%)
HB VI at last follow-up	2/19 (10.5%)
Hearing preservation after revision	0/19 (0%)
Further regrowth during follow-up	1/19 (5.3%)
Radiological tumor control at last follow-up	18/19 (94.7%)

During follow-up, further tumor regrowth occurred in 1/19 patients (5.3%), in whom a small residual had been intentionally left due to brainstem adherence; no further surgery was indicated because of the patient’s overall clinical condition. Thus, radiological tumor control at last follow-up was 18/19 (94.7%).

## Discussion

Revision microsurgery for VS represents one of the most technically challenging scenarios in skull base surgery. Irrespective of the initial treatment modality—prior microsurgery, stereotactic irradiation, or combined approaches—revision cases are characterized by distorted anatomy, fibrosis, obliterated arachnoid planes, and frequent adherence of tumor capsule to cranial nerves, brainstem, and vascular structures. These factors typically translate into linferior functional outcomes compared with primary surgery and necessitate a tailored, risk-adapted surgical strategy with explicit preoperative counseling.

### Principal findings

In our consecutive cohort of 19 revision procedures, most tumors were advanced at re-presentation (Koos grade 4 in 74%), and revision surgery was predominantly performed via a retrosigmoid–transmeatal approach. We achieved GTR in 78.9% of cases and favorable long-term facial nerve function (HB I–III) in 68%. Hearing was not preserved in any patient after revision surgery. Importantly, subsequent tumor regrowth after revision was observed in only one patient in whom a small residual was intentionally left due to brainstem infiltration. When placed in the context of published revision series (Table 5), our rates of macroscopic tumor clearance and favorable facial outcomes fall within the range reported by high-volume centers, despite a predominance of large tumors at presentation.

**Table 5:** Summary of key published revision series

Study	Patients (*n*)	Prior treatment	Revision approach	GTR	Favorable facial outcome	FN metric/definition	Follow-up	Tumor control / retreatment
Peng et al. 2018 [12]	250	Mixed (post-surgery and post-RT)	Predominantly translabyrinthine	212/250 (84.8%)	HB I–III: 116/250 (46.4%)	HB at last follow-up	Mean 53 mo	11/250 retreatment (7 SRS, 4 surgery)
Przepiórka et al. 2020 [13]	29	Post-surgery only	TLA 14; RSA 12; combined 2; MF 1	29/29 (100.0%)	HB I–III: 19/29 (65.5%)	HB at last follow-up	Mean 3.46 y	No recurrences reported
Perry et al. 2017 [14]	6	Post-surgery only (after intended GTR)	Alternative approach in most cases	4/6 (66.7%)	HB I–II: 5/6 (83.3%)	HB I–II at last follow-up	Mean 20 mo (range 5–36)	Not clearly reported
Samii et al. 2016 [15]	53	Mixed (prior surgery only; prior surgery + RT)	Retrosigmoid (all cases)	53/53 (100.0%)	HB I–II: 34/53 (64.2%)	HB I–II at 1 year	Mean 5 y (up to 11 y)	No recurrences reported
Freeman et al. 2007 [16]	27 pts/35 ops	Post-surgery failures (residual/recurrent)	Mixed	14 total excisions (ops-level)	HB I–III: 63% (authors)	Ops vs. pts; heterogeneous	Long-term (variable)	1 recurrence after total excision (reported)

Collectively, these findings suggest that in a high-volume tertiary setting, revision microsurgery can provide high rates of macroscopic tumor clearance and satisfactory long-term facial nerve outcomes in a substantial subset of patients, albeit at the expected cost of inferior functional outcomes compared with primary surgery [17].

### Revision surgery is uncommon, and indication must be stringent

A critical implication of contemporary VS management is that radiological persistence of small remnants does not automatically mandate reintervention. Large registry-based and institutional series demonstrate that many small remnants remain stable or may even become non-visible on MRI, likely due to devascularization; only a minority of patients require revision surgery or additional radiotherapy [18]. This observation is central to treatment planning: the decision to offer revision surgery should be driven by well-documented and sustained growth (and/or progressive symptoms), rather than by the mere presence of postoperative enhancement or minimal residual disease.

Our low frequency of revision surgery (2.6% of all treated VS; 1.1% within our own primary microsurgical cohort) is consistent with this framework and reinforces the concept that, for many patients, long-term surveillance and selective salvage is a rational pathway rather than immediate aggressive reoperation. Published evidence on revision microsurgery remains limited and heterogeneous, reflecting the rarity of the clinical scenario and substantial variability in definitions (recurrence vs. regrowth), endpoints, follow-up duration, and prior treatment modalities. Nonetheless, several reference series provide context.

Freeman et al. reported outcomes of revision surgery for residual/recurrent VS in a large tertiary service, showing that revision operations frequently resulted in less-than-total excision and that additional treatment was often required after near-total and subtotal revisions, highlighting the challenge of achieving durable control in revision settings [16].

Peng et al. published the largest contemporary revision series to date (250 revision surgeries), reporting GTR in 85% and mean postoperative facial nerve function around HB 3.8–3.9 at last follow-up, with a slightly increased CSF leak rate compared with primary surgery [12]. Their cohort predominantly utilized the translabyrinthine approach, whereas our revision strategy was mainly retrosigmoid–transmeatal, reflecting institutional preference and/or tumor morphology and extension.

For the specific and relatively rare scenario of repeat microsurgery after a prior intended GTR, Perry et al. reported GTR in 67% and excellent facial function (HB I–II) in 87% in a small series, and emphasized that choosing an alternative approach at repeat surgery may reduce scar-related dissection challenges [14].

Regarding the impact of prior irradiation, multiple authors note that salvage microsurgery after failed stereotactic irradiation can be particularly demanding and may require acceptance of less-than-complete resection to preserve facial nerve integrity [19]. Hong et al. similarly concluded that outcomes in recurrent VS are generally less favorable than in primary surgery and discussed the unresolved question of whether prior surgery versus prior radiation differentially affects postoperative facial nerve outcomes [20].

Against this background, our rates of GTR (79%) and HB I–III (68%) are within the range of published revision experiences, while our absence of hearing preservation is not unexpected given that most patients were already non-serviceably hearing or deaf preoperatively and that revision goals prioritize tumor control and facial nerve management.

### Facial nerve outcomes and reconstructive strategy

Facial nerve function is the dominant driver of long-term quality of life after VS treatment, and revision surgery introduces additional risk because the nerve may be thinned, displaced, or directly incorporated into scar and tumor capsule [1]. In our cohort, facial nerve discontinuity was identified intraoperatively in nearly half of the cases, necessitating reconstructive procedures (predominantly XII–VII anastomosis using the Darrouzet modification, and less frequently interposition grafting) [21]. While immediate postoperative HB VI was common, most patients improved over time, leaving only 11% with persistent HB VI at last follow-up.

These data support two practical points: (i) revision surgery should be conceptualized not only as a tumor operation but frequently as a combined tumor-and-facial-nerve procedure; and (ii) centers managing revision VS should have an established algorithm for intraoperative decision-making and postoperative facial reanimation pathways. From the counseling perspective, patients should be informed that even when the nerve is anatomically preserved, functional recovery is less predictable than in primary surgery; conversely, when the nerve is disrupted, meaningful long-term improvement remains achievable but depends on prompt reconstruction and rehabilitation.

### Tumor control, planned residuals, and long-term follow-up

Our tumor control after revision was high, with only one regrowth observed during extended follow-up occuring in a patient with a deliberate residual due to close brainstem adherence. This observation is consistent with the broader concept that small remnants may remain stable and that not all radiologically detectable disease is biologically progressive [18]. However, it also underlines the importance of clearly defined radiological criteria of progression in post-treatment settings—particularly after stereotactic irradiation where transient enlargement and treatment-related changes may confound early imaging interpretation.

Clinically, these considerations argue for a structured follow-up strategy with predefined imaging intervals and specific growth criteria that trigger reintervention. In revision candidates with borderline indications, a short-interval MRI reassessment may be preferable to immediate salvage surgery, provided symptoms and brainstem compression are controlled.

### Limitations

This study has the limitations inherent to a retrospective single-center design.

First, the cohort size is small, reflecting the rarity of revision surgery even at high-volume tertiary centers — a finding that is itself consistent with contemporary outcome-focused VS management, in which the majority of postoperative remnants remain stable under surveillance and do not require reintervention. While this limits statistical power and precludes multivariable analysis, we note that the consecutive enrollment, histopathological verification of all included cases, and mean follow-up of 136 months represent methodological strengths that provide complementary value to larger but shorter-followed series in the existing literature.

Second, the long inclusion period (1998–2025) likely introduced heterogeneity in microsurgical technique, intraoperative monitoring, imaging quality, and radiosurgical protocols. Third, although facial nerve function and audiometry are standardized, the timing of “final” functional assessment and the completeness of longitudinal follow-up may vary. Finally, we did not include patient-reported outcome measures, which are increasingly considered essential to fully capture the functional impact of treatment.

### Clinical implications

Within contemporary outcome-oriented VS management, revision microsurgery should be framed as a highly selective salvage modality reserved for clearly progressive or symptomatic disease. The decision to operate should incorporate (i) objective evidence of sustained growth, (ii) tumor size and brainstem compression, (iii) baseline cranial nerve status, and (iv) the anticipated probability of achieving durable control without unacceptable morbidity. When performed in experienced centers with established facial nerve reconstruction capability, revision surgery can achieve high rates of tumor clearance and clinically meaningful facial nerve outcomes; nevertheless, expectations must be explicitly calibrated to the revision setting.

## Conclusion

Revision microsurgery for VS is uncommon but remains an indispensable component of salvage management. In our cohort, high rates of radical resection and favorable long-term facial nerve outcomes were achievable despite a predominance of large tumors at re-presentation, while hearing preservation was not attainable. These results support revision surgery as an effective option for selected patients with confirmed progression after prior treatment, particularly in high-volume centers with comprehensive cranial nerve expertise.

## Data Availability

Data available from the corresponding author on reasonable request.
